# Publications Received

**Published:** 1858-11

**Authors:** 


PUBLICATIONS RECEIVED.

"Uremic Convulsions of Pregnancy, Parturition, and Child-bed." By Dr. Carle R. Braun, Prof. of Midwifery, Vienna. Translated from the German, with notes by J. Matthews Duncan, F. R. C. P. E., Lecturer on Midwifery, &c. This is an extract in book form, (from the press of Samuel S. & W. Wood,) from a new work published by Dr. Braun.

It is regarded as a valuable addition to our knowledge upon this subject, and the whole work will be expected with much anxiety.

---

"Annual Report and Circular of the New Orleans School of Medicine."

From this Report, we learn that the Institution is now perfectly equipped in every respect; its condition prosperous, and its prospects brilliant. At the close of their first lecture term (last year) they reported a list of 76 matriculates, and 26 graduates; at the close of the 2d term, just passed, they report 126 matriculates, and 34 graduates.

The Faculty express the opinion, that the establishment of a second Medical School in New Orleans was demanded, and give as one evidence, the increase in the number of matriculates in the City from 230 to 400, and predict that in five years the number of Students attending lectures in N. Orleans will be 7 or 800. The City of New Orleans possesses great advantages for medical instruction. Success to both Schools.

